# Effects of Pr and Yb Dual Doping on the Thermoelectric Properties of CaMnO_3_

**DOI:** 10.3390/ma11101807

**Published:** 2018-09-23

**Authors:** Cuiqin Li, Qianlin Chen, Yunan Yan

**Affiliations:** 1 School of Chemistry and Chemical Engineering, Guizhou University, Guiyang 550025, China; licq2345@163.com; 2 National & Local Joint Laboratory of Engineering for Effective Utilization of Regional Mineral Resources from Karst Areas, Guiyang 550025, China; 3 School of Physics and Optoelectronic Engineering, Ludong University, Yantai 264025, China; yunan@ldu.edu.cn

**Keywords:** dual doping, CaMnO_3_, coprecipitation method, thermoelectric properties

## Abstract

There has been research on CaMnO_3_ with natural abundance, low toxicity, and low cost as promising candidates for n-type thermoelectric (TE) materials. In this paper, Ca_1−2x_Pr_x_Yb_x_MnO_3_ with different Pr and Yb contents (x = 0, 0.01, 0.02, 0.03, 0.04 and 0.05) were synthesized by means of coprecipitation. With X-ray diffraction (XRD), scanning electron microscopy (SEM), and high-resolution transmission electron microscopy (HRTEM), researchers characterized the phase structure and morphology of all the samples. The oxidation states of manganese were determined by X-ray photoemission spectroscopy (XPS). The role of Ca-site dual doping in the TE properties was also investigated. Increasing the Pr and Yb contents leads to decreases in the electrical resistivity and Seebeck coefficient, leading to a power factor of 3.48 × 10^−4^ W·m^−1^·K^−2^ for x = 0.04 at 773 K, which is its maximum. Furthermore, the thermal conductivity (κ) decreases with increasing x, and *κ* = 1.26 W·m^−1^·K^−1^ is obtained for x = 0.04 at 973 K. Ca_0.92_Pr_0.04_Yb_0.04_MnO_3_ exhibit a ZT (thermoelectric figure of merit) value of 0.24 at 973 K, approximately 3 times more than that of the pristine CaMnO_3_. Thus, the reported method is a new strategy to enhance the TE performance of CaMnO_3_.

## 1. Introduction

The rapid growth and substantial increase in human energy demand has become a global problem of the twenty-first century. Reserves of fossil fuels such as coal, petroleum and natural gas are becoming increasingly depleted. Improvements in the comprehensive utilization of existing technologies, the development of current energy sources and the identification of new renewable energy sources are urgently needed [1]. Due to the generation of large amounts of waste heat across the world whichpollutes the environment, the conversion of waste heat into useful energy is an attractive target. Thermoelectric (TE) devices are considered promising candidates because they can directly convert waste heat into electrical energy [2]. Both n-type and p-type TE materials are superior in terms of simplicity, safety, stability, lack of moving parts, and no maintenance, leading to widespread interest in recent years [3,4].

The energy conversion efficiency of TE materials is calculated by a figure of merit, ZT (=S^2^T/*ρκ*), in which S refers to the Seebeck coefficient, *ρ* refers to the electrical resistivity, T refers to the working temperature in Kelvin, and *κ* refers to the thermal conductivity [5]. Both a high-power factor (PF) (S^2^/*ρ*) and a low value of *κ* are required for practical use. Recently, improvements at the proof-of-concept level combined with advanced synthetic methods have resulted in alloy TE materials with ZT values higher than 2 [6,7,8,9,10]. However, the disadvantages of high cost, toxicity and weak thermal stability severely restrict the application of these alloys as good TE materials. Therefore, oxide thermoelectric materials as promising high-temperature materials with structural and chemical stability, oxidative resistance, environmental friendliness and low cost have received increasing attention. Among the oxide TE materials, n-type CaMnO_3_ have received increasing attention owing to their high Seebeck coefficient (−550 μV/K) [11]. The electrical resistivity and thermal conductivity of these oxide materials, however, are too high for TE applications. Numerous efforts, such as doping the Ca site with different high-valent cations, have been devoted to improving the TE properties of CaMnO_3_. The effect of high-valent cation addition on the TE properties of CaMnO_3_ was first investigated by M. Ohtaki et al. The researchers found that Ca_0.9_Bi_0.1_MnO_3_ exhibited a maximum PF of 2.8 × 10^−4^ W·m^−1^·K^−2^ at 1073 K and showed a large ZT value of 0.085 [12]. Based on that study, the introduction of high-valent lanthanides at the Ca site was frequently reported to increase the charge carrier concentration through forming Mn^3+^ in the Mn^4+^ matrix, which is in accordance with electron doping in the eg states. Moreover, the enormous mass difference between rare-earth (RE)^3+^ and Ca^2+^ ions was expected to reduce the thermal conductivity. Therefore, the conversion efficiency improved. Substitution with lanthanides at the Ca site was reported by D. Flahaut et al., and Y. Wang et al. For samples with lanthanide doping concentrations higher than 10% at the Ca site prepared through a solid-state method, the resistivity, which was found to depend on the RE size, exhibited a minimum for Yb substitution [13,14,15], whereas the absolute values for both the Seebeck coefficient and the thermal conductivity decreased relative to those of undoped CaMnO_3_, with maxima of ZT = 0.16 and ZT = 0.2, respectively, at T = 1000 K for Ca_0.9_Yb_0.1_MnO_3_. Substitution of Pr^3+^ in Ca_1−x_Pr_x_MnO_3_ was also reported to enhance the TE performance, with ZT = 0.16 at 873 K for x = 0.08 [16] or at 1100 K for x = 0.14 [17]. However, dual doping of the Ca sites of CaMnO_3_ has proven to be more effective at promoting TE properties [18,19,20]. The TE performance of CaMnO_3_ dual-doped with R^3+^ elements was originally proposed by A. Kosuga et al. [21], while subsequent studies of doping with other R^3+^ ions were carried out by C.M. Kim and H.C. Wang [22,23]. The researchers found that the resistivity and Seebeck coefficient were both reduced by the introduction of multiple elements at the Ca site. In addition, the thermal conductivity, which was affected by both crystal distortion and the tremendous difference in mass between Ca^2+^ and R^3+^ ions, was also reduced [21]. All the CaMnO_3_ mentioned above were synthesized through a solid-state method. However, the coprecipitation method exhibits many advantages over the conventional solid-state method. The mixed nitrate precursors are dissolved in water in advance, enabling the dopants to be better dispersed in matrix materials and powder, leading to a narrow particle distribution and microstructural homogeneity of the sintered product. Romy Löhnert et al., found that Ca_0.96_Gd_0.04_MnO_3_ synthesized by a coprecipitation method had a narrow particle distribution and small particle size, and they exhibited a much lower thermal conductivity than prepared by the conventional solid-state method. The highest observed ZT value was 0.14, which was found at 973 K for the Ca_0.96_Gd_0.04_MnO_3_ prepared by the coprecipitation method; this value was 1.5 times larger than the one obtained for the sample produced through the solid-state method [24].

In the present paper, Ca_1−2x_Pr_x_Yb_x_MnO_3_ with different Pr and Yb contents (x = 0, 0.01, 0.02, 0.03, 0.04 and 0.05) were fabricated for the first time by coprecipitation. The effects of Pr and Yb substitution on the microstructure and TE properties were investigated. Possible mechanisms were also explored. In addition, the effects of Pr and Yb dual doping on a CaMnO_3_ system density of states (DOS) were investigated using first-principles calculations to better understand the TE properties of the Pr and Yb dual-doped CaMnO_3_ system.

## 2. Materials and Methods

### 2.1. Sample Synthesis

Polycrystalline samples of Ca_1−2x_Pr_x_Yb_x_MnO_3_ (x = 0, 0.01, 0.02, 0.03, 0.04 and 0.05) were prepared by a coprecipitation process in which Ca(NO_3_)_2·_4H_2_O (purity 99.95%), Mn(NO_3_)_2_ solution (50%, *w*/*w*), Yb(NO_3_)_3_·6H_2_O, Pr(NO_3_)_3_ and NH_4_HCO_3_ were used as raw materials. Ca(NO_3_)_2_, Mn(NO_3_)_2_ solution, Pr(NO_3_)_3_ and Yb(NO_3_)_3_ powder were mixed in stoichiometric ratios and dissolved in distilled water to prepare the nitrate stock solution. Next, excess NH_4_HCO_3_ (purity 99.99%) as a precipitation agent (20%, *w*/*w*) was added dropwise to the nitrate solution with continuous stirring and moderate heating until pH > 8.5 to completely precipitate the metal ions. The resultant suspension was subjected to suction filtration, and the collected precipitate was washed with distilled water many times before being dried at 343 K for 2 h. Then, the resultant precursor powder was calcined in air at 1173 K for 8 h, forming a complex precursor powder of oxide. The obtained powder was pelletized with pressure and sintered in air at 1523 K for 48 h with intermediate grinding to ensure the absence of precursor phases.

### 2.2. Characterization

The phase purity of the powder samples after sintering was studied by means of X-ray diffraction (XRD, PANalytical B.V., X’Pert PRO, Almelo, The Netherlands) with Cu Kα radiation (1.5406 Å). XRD data were obtained at 0.02° intervals between 15° and 120°. Quantitative Rietveld refinement was carried out using the FullProf program [25] for profile analysis based on the crystal structure of pure CaMnO_3_ proposed by H. Taguchi [26], and marokite CaMn_2_O_4_ was included as an additional phase, as proposed by H.G. Giesber [27]. In the refinement process, the peak reflection shapes were modelled through a pseudo-Voigt profile function corrected for asymmetry and the background by way of a linear interpolation. The refinement data were obtained by refining the following parameters: scale factors, background parameters, zero-point error, lattice parameters, half-width parameters (U, V and W), atomic position parameters, peak width parameters, peak shape parameters and profile asymmetry parameters [28]. The powder samples were digested with Aqua regia, then the content of Pr^3+^and Yb^3+^ were determined by using an inductively coupled plasma atomic emission spectrometry (Avio 200, PerkinElmer, Waltham, MA, USA). X-ray photoelectron spectroscopy (XPS) measurements were carried out on an ESCA-LAB250Xi (Thermo Scientific, Waltham, MA, USA) spectrometer with Al Kα_1,2_ photons. A Shirley background correction was used for all spectra. Additionally, the morphology was obtained through scanning electron microscopy (SEM, JSM-6460LV, JEOL, Tokyo, Japan). To further investigate the local structure of the dual-doped particles, high-resolution transmission electron microscopy (HRTEM, Tecnai G2 F20 S-TWIN, FEI, Hillsboro, OR, USA) observations were carried out.

### 2.3. Thermoelectric Properties

The pellets obtained after sintering were chopped into pieces with a dimension of 10 mm × 2 mm × 2 mm for simultaneous measurements of electrical resistivity and Seebeck coefficient using an UlvacRiko ZEM-3 system (Yokohama, Japan). The disc-shaped pellets were all polished to a diameter of 12.7 mm and a thickness of 2 mm to determine both the thermal diffusivity (D) with a business system (LFA-447 Nanoflash, Netzsch, Selb, Germany) and the designated heat capacity (C_p_) with commercial equipment (DSC 200 F3, Netzsch, Selb, Germany). The thermal conductivity was computed with the following formula: *κ* = DC_p_da. The actual density (da) of the sample pellets was obtained in accordance with the Archimedes method.

### 2.4. Computational Method

The first-principles spin-polarized calculations were carried out within the CP2K/Quickstep package [29]. As density functional theory methods tend to underestimate the band gap, we adopted Hubbard U correction throughout the calculations [30]. The U values corresponding to the Hubbard U correction of Mn 3d and O 2p orbitals were designated as 3.9 eV and 3.5 eV, respectively. With the norm-conserving Goedecker–Teter–Hutter (GTH) pseudopotentials, the core electrons of all elements were described [31]. With the use of the Gaussian function with molecularly optimized double-zeta polarized basis sets (m-DZVP), the wave functions of Ca 3s^2^3p^6^4s^2^, Mn 3s^2^3p^6^3d^5^4s^2^, O 2s^2^2p^4^, Pr 4f^3^5s^2^5p^6^6s^2^, and Yb 5s^2^5p^6^6s^2^4f^14^ electrons were described [32]. A 500 Ry cut-off energy was applied as the auxiliary basis set for plane waves. With regard to the calculation structures, a supercell of CaMnO_3_ (a = 10.67 Å, b = 7.57 Å, c = 10.82 Å) was used. To simulate the Pr and Yb dual-doped structure, different concentrations of Ca atoms were substituted by the RE elements Pr and Yb. The Fermi level was set as the zero point in all relative calculations.

## 3. Results

### 3.1. Characterization of Structure and Composition

**Figure 1:**
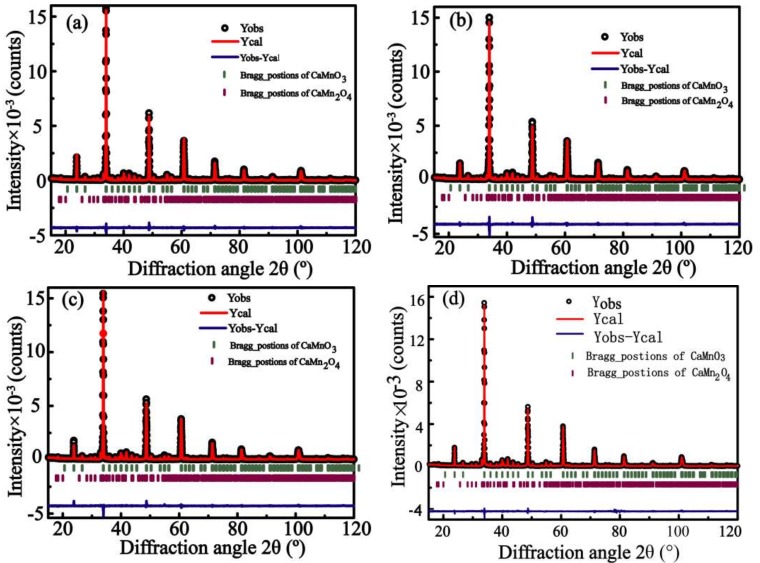
Observed (black circles) and calculated (red line) X-ray diffraction (XRD) patterns together with their difference curve (bottom line) after Rietveld refinement (*λ* = 1.4506 Å) for Ca_1−2x_Pr_x_Yb_x_MnO_3_. The upper reflection pattern corresponds to CaMnO_3_, and the lower pattern corresponds to CaMn_2_O_4_. (**a**) x = 0; (**b**) x = 0.01; (**c**) x = 0.03; (**d**) x = 0.05.

**Figure 2:**
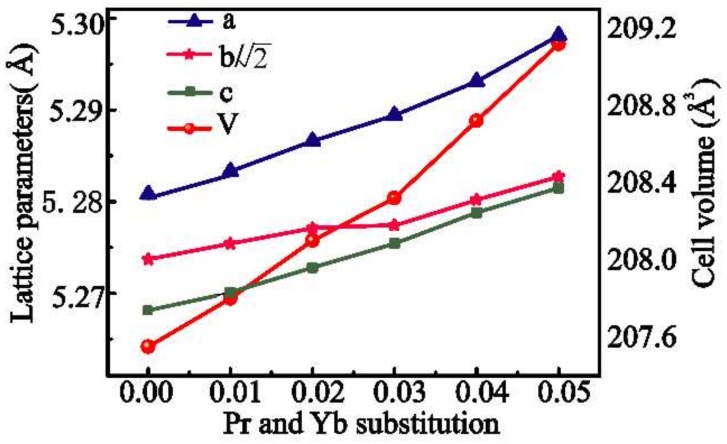
Pr and Yb dual-doping dependence of the lattice parameters and cell volumes of the Ca_1−2x_Pr_x_Yb_x_MnO_3_ series.

In Figure 1, the refined XRD patterns of the Ca_1−2x_Pr_x_Yb_x_MnO_3_ (x = 0, 0.01, 0.03, 0.05) powders obtained by the coprecipitation method are presented. The main phase observed for all the samples at 298.15 K can be assigned to orthorhombic CaMnO_3_ (pdf #89-0666, space group Pnma 62). A small amount of CaMn_2_O_4_ is detected (2–5%) as a secondary phase in all the studied samples. The refined structural parameters, bond distances, distortion angles, and tolerance factor (t) of the Ca_1−2x_Pr_x_Yb_x_MnO_3_ (x = 0, 0.01, 0.02, 0.03, 0.04 and 0.05) series are summed up in Table 1. The relationships between the lattice parameters, cell volume and the dual-doping composition x are shown in Figure 2. Table 1 and Figure 2 show that with an increase in the concentration of Pr and Yb, the lattice parameters and cell volume increase monotonically, which may be illustrated through the fact that although the ionic radius of the Yb^3+^ doping ions (0.985 Å) is smaller than that of Ca^2+^ (1.12 Å), the ionic radius of the Pr^3+^ doping ions (1.126 Å) is a litter bit larger than that of Ca^2+^. Furthermore, with the introduction of higher valence states Pr^3+^ and Yb^3+^ at the Ca site, Mn^3+^ presence is induced within the Mn^4+^ matrix, corresponding to charge compensation principle, and the ionic radius of Mn^3+^ exceeds that of Mn^4+^ (r[Mn^4+^] = 0.530 Å and r[Mn^3+^] = 0.645 Å) [33]. So, the increasing Pr^3+^ and Yb^3+^ concentration together with the increasing value of Mn^3+^/Mn^4+^ expand the lattice parameters and cell volume. This phenomenon has also been documented by Zhu et al. [34]. The following relation is also observed in Table 1 and Figure 2: c < b/$\sqrt{2}$ < a, indicating the occurrence of the deformation of O-type orthorhombic of Ca_1−2x_Pr_x_Yb_x_MnO_3_ phases, which is consistent with the results of a previous report [35].

**Figure 3:**
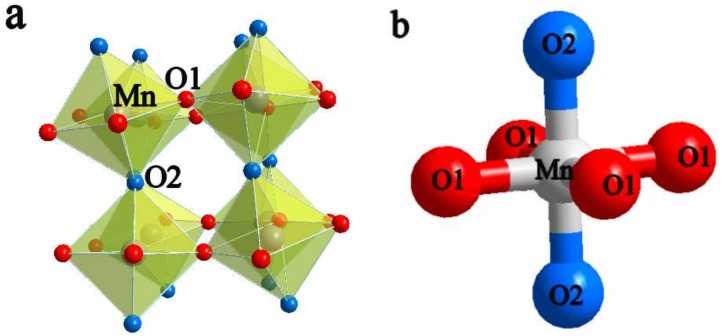
(**a**) Distorted MnO_6_ octahedron; (**b**) geometric structure of the distorted octahedron of Ca_0.90_Pr_0.05_Yb_0.05_MnO_3_.

In orthorhombic perovskite-type Ca_1−2x_Pr_x_Yb_x_MnO_3_ (x = 0, 0.01, 0.02, 0.03, 0.04 and 0.05), the A-site cations (Ca, Pr and Yb ions) are coordinated to 12 anions: 8 O (1) and 4 O (2) ions. The B-site cations (Mn ions) are coordinated to 6 anions: 4 O (1) (a–c plane) and 2 O (2) ions (b axis) [36]. As seen from Table 1, the 4 Mn-O1 bond distances and 2 Mn-O2 bond distances increase with increasing Pr and Yb dual-doping content, in which the increase in the 4 Mn-O1 bond distances is larger than that in the 2 Mn-O2 bond distances because of the JahnTeller effect induced by Mn^3+^ [37]. This effect causes changes in the lattice parameters and eg electrons in the dz^2^ orbital and a–c plane of the Mn^3+^ centres of the MnO_6_ octahedron. The tolerance factor, t, quantitatively describes the geometrical distortion of the structure, where t = 1 represents a perfect structure of ABO_3_ perovskite. With regard to the Pr and Yb dual-doped phases, the tolerance factor decreases with increasing Pr and Yb substitution, indicating that the distortion of the MnO_6_ octahedron (The distorted MnO_6_ octahedron and geometric structure of distorted MnO_6_ octahedron for Ca_0.90_Pr_0.05_Yb_0.05_MnO_3_ are listed in Figure 3a,b) is intensified in this structure, along with the fact that the Mn-O-Mn angles measured are unequal to 180°. The angles of the Mn-O2-Mn bond decrease, making the band gap energy of the perovskite crystal structure change, narrowing the eg electron conduction bandwidth and resulting in improved electrical properties [38]. This result is demonstrated by the following calculated electronic structure of the dual-doped CaMnO_3_ series.

Table 2 shows the results of chemical analysis, and density measurement. The theoretical density (dt) of a given sample was calculated using the following express: dt = 4 MW/N_A_V, where MW, V, N_A_ is the molecular weight, the cell volume V (obtained from refinement) and the Avogadro’s constant, respectively.

**Figure 4:**
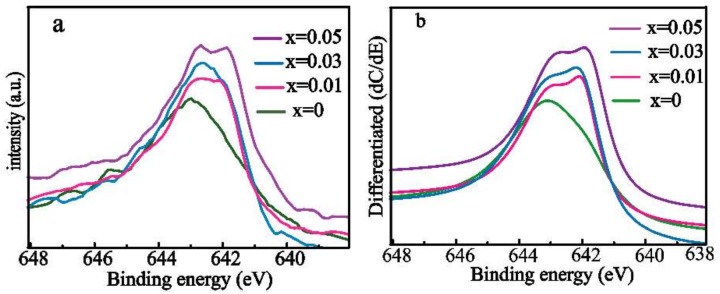
(**a**) Mn 2p3/2 XPS spectra; (**b**) differentiated spectra for the Ca_1−2x_Pr_x_Yb_x_MnO_3_ (x = 0, 0.01, 0.03, and 0.05) series.

Figure 4a shows the XPS data for the Mn 2p3/2 oxidation states of Ca_1−2x_Pr_x_Yb_x_MnO_3_ (x = 0, 0.01, 0.03 and 0.05). Y.G. Wei and colleagues proposed that the Mn 2p3/2 XPS binding energies for Mn^3+^ and Mn^4+^ are 641.9 and 643.2 eV, respectively [39]. To highlight these spectral differences, differentiated XPS spectra were also obtained and are shown in Figure 4b, in which the Mn^3+^ peak areas systematically increase with increasing Pr and Yb concentration in Ca_1−2x_Pr_x_Yb_x_MnO_3_ (x = 0, 0.01, 0.03 and 0.05). This trend shows that the concentration of Mn^3+^ increases with increasing Pr and Yb dual doping. The obtained results show good agreement with the XRD results discussed above.

**Figure 5:**
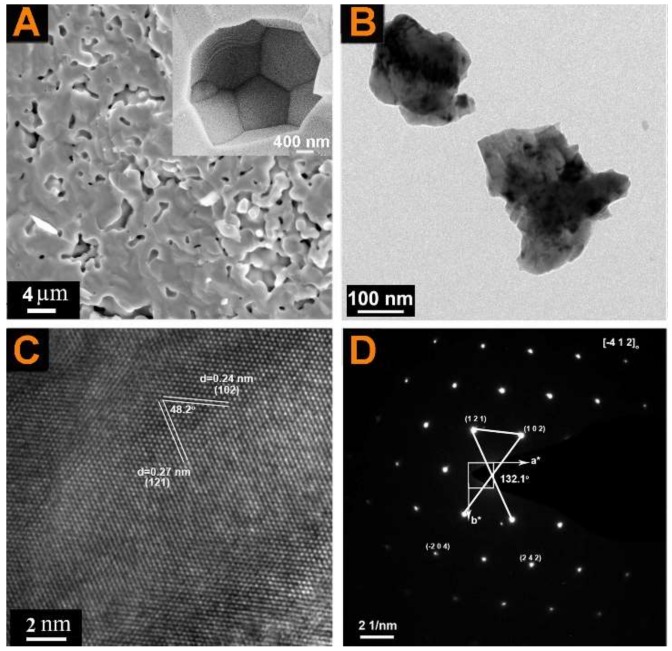
SEM and TEM images of Ca_0.90_Pr_0.05_Yb_0.05_MnO_3_: (**A**) SEM image of bulk; (**B**) low-magnification TEM image of powder; (**C**) high-magnification TEM image of powder; and (**D**) electron diffraction pattern of powder.

Figure 5A exhibits the typical cross-section morphology of sintered pellets of Ca_0.90_Pr_0.05_Yb_0.05_MnO_3_ obtained by the coprecipitation process. The same particle morphology with a narrow average particle size distribution is presented in all samples. Grains 1–5 µm in size are achieved for the coprecipitation phases in all the samples, while the grain size of the solid-state reaction-derived phases is 3–10 µm [35,40], indicating that the coprecipitation process can be applied to prepare fine-grained materials. Meantime, it is found that the Pr and Yb dual-doped samples are denser than the undoped sample. Table 2 presents the relative density of all the samples. The relative density of all the Pr and Yb dual-doped bulk samples is higher than that of undoped sample, indicating that Pr^3+^ and Yb^3+^ be used as sintering aid to enhance the density of the Pr and Yb dual-doped samples. The HRTEM pattern shows well-resolved interference fringe spacings of 0.24 nm and 0.27 nm, which agree well with the interplanar distances of the 102 lattice planes and 121 lattice planes, respectively (d_102_ = 2.04 Å and d_121_ = 2.07 Å based on the powder XRD card 89-0666).

### 3.2. Thermoelectric Properties

**Figure 6:**
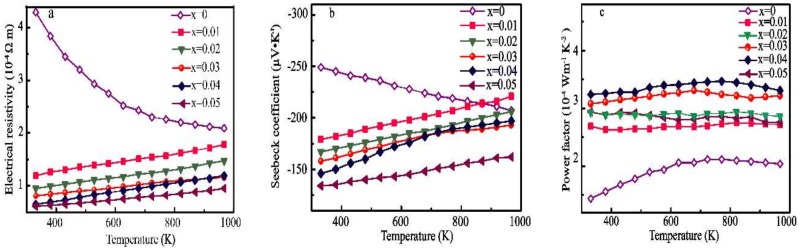
Temperature dependence of (**a**) the electrical resistivity, *ρ*; (**b**) the Seebeck coefficient, S; and (**c**) the power factor, PF, for all the samples.

Figure 6a illustrates the temperature dependence of *ρ* for the Pr and Yb dual-doped Ca_1−2x_Pr_x_Yb_x_MnO_3_ (x = 0, 0.01, 0.02, 0.03, 0.04 and 0.05) bulk under temperatures in the range of 300–1000 K. The *ρ* value of pristine CaMnO_3_ is initially 4.28 × 10^−4^ Ω m and decreases to 2.09 × 10^−4^ Ω m as the temperature increases, showing typical semiconductor with *∂ρ*/*∂*T < 0. However, the dual doping of Pr and Yb at the Ca site dramatically lowers the ρ value in the CaMnO_3_ system. The *ρ* curve temperature dependence of the five dual-doped samples exhibit characteristics of metallic, with *∂ρ*/*∂*T > 0, in which the electrical resistivity increases with increasing temperature. Similar is also found in the single Pr- and Yb-doped CaMnO_3_. The value of *ρ* decreases gradually with increasing Pr and Yb dual-doping concentrations. The Ca_0.9_Pr_0.05_Yb_0.05_MnO_3_ show the lowest electrical resistivity.

Which is smaller than those of the Ca_0.90_Pr_0.1_MnO_3_ and Ca_0.90_Yb_0.1_MnO_3_ made through the solid-state process [13,17], confirming that coprecipitation dual doping is more effective than single doping of Pr or Yb at the Ca site. The significant reduction in the dual-doped samples’ electrical resistivity can be attributed to the Mn^3+^ (t^3^_2g_e^0^_g_) cations forming in the Mn^4+^ (t^3^_2g_e^1^_g_) matrix of CaMnO_3_ due to dual doping by high-valent Pr^3+^ and Yb^3+^ cations at the Ca site, which is further supported by analyses of the above crystal structure and XPS data. An appreciable concentration of Mn^3+^ is formed as a result of Pr^3+^ and Yb^3+^ dual doping at the Ca site, introducing free electrons into the eg orbital. In the eg level, the electrons function as charge carriers in the Mn^3+^-O-Mn^4+^ framework, reducing ρ with their movement through a hopping mechanism.

The temperature dependence of the Seebeck coefficient of the Ca_1−2x_Pr_x_Yb_x_MnO_3_ (x = 0, 0.01, 0.02, 0.03, 0.04 and 0.05) is presented in Figure 6b. The Seebeck coefficient is negative for all samples, indicative of n-type conductive with a dominant electrical carrier. The results demonstrate that the Seebeck coefficient for all the samples possesses a similar dependence on negative temperature. The pristine CaMnO_3_ show a large Seebeck coefficient, which decreases with increasing temperature as the result of the low carrier concentration of the pristine material. The substitution of the divalent Ca^2+^ cation by trivalent Pr and Yb ions induces the formation of Mn^3+^ with an eg1 configuration as an electron carrier, resulting in a lower absolute Seebeck coefficient. The absolute Seebeck coefficient can be calculated by the following model proposed for degenerate semiconductors [41]:

(1) $$S=\frac{8\pi^{2}k_{B}^{2}}{3eh^{2}}m\cdot T\;{\left(\frac{\pi }{3n}\right)}^{\frac{2}{3}}$$

where *k_B_* is the Boltzmann constant and *n* and m^*^ refer to the carrier concentration and the effective mass of the carrier, respectively. The absolute Seebeck coefficient values are inversely proportional to *n*. The Seebeck coefficient decreases with increasing Pr and Yb dual-doping concentration, which is attributed to increasing *n*. The absolute value of the Seebeck coefficient gradually decreases when the dual-doping concentration increases, which is in line with the decreasing trend in the electrical resistivity. The absolute Seebeck coefficient decreases from S_973K_ = 221 µV·K^−1^ (x = 0.01) to S_973K_ = 162 µV·K^−1^ (x = 0.05) along with an increase in the Pr and Yb dual-doping concentration.

The PF values of all the samples were obtained through the above *ρ* and S values and are plotted as a function of temperature in Figure 6c. The PF variation in all the samples as a function of temperature is similar to that in *ρ*. In all the samples, PF increases significantly with the increase in the temperature due to the decreased *ρ* and increased Seebeck coefficient. The PF is inversely proportional to *ρ* and proportional to the square of the Seebeck coefficient. The trend in the PF in all the samples is approximately similar to that in *ρ*. Initially, PF increases to reach a maximum at x = 0.04 and 973 K and then decreases. The largest PF of 3.48 × 10^−4^ W·m^−1^·K^−2^ at 773 K is obtained for Ca_0.92_Pr_0.04_Yb_0.04_MnO_3_; this value is approximately two times higher than that obtained for the pristine CaMnO_3_. Compared to other doped CaMnO_3_, the PFs obtained in the Pr and Yb dual-doped CaMnO_3_ prepared by the coprecipitation method are moderate but much higher than those of the Pr or Yb single-doped CaMnO_3_ made through the solid-state process.

**Figure 7:**
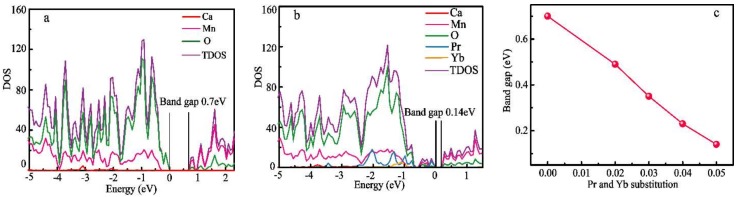
The DOS of (**a**) CaMnO_3_; (**b**) Ca_0.90_Pr_0.05_Yb_0.05_MnO_3_; and (**c**) The dependence of the band gap on the Pr and Yb dual-doping concentration.

The transport properties of a semiconductor are well known to be dominated by the electronic structure close to the Fermi level [6,42]. The theoretically calculated DOS of CaMnO_3_ and Ca_0.90_Pr_0.05_Yb_0.05_MnO_3_ are displayed in Figure 7a,b. The theoretically calculated band gap of the CaMnO_3_ is 0.70 eV, in accordance with the values of previous reports [43,44], indicating that the calculation parameters are reasonable. The dependence of the band gap on the Pr and Yb dual-doping concentration is illustrated in Figure 7c. The band gap of the Pr and Yb dual-doped CaMnO_3_ samples decrease with the increasing Pr and Yb dual-doping amount. For the CaPr_0.05_Yb_0.05_MnO_3_, the theoretically calculated band gap is 0.14 eV, which is much lower than that obtained in undoped CaMnO_3_. The band gap of the Pr and Yb dual-doped CaMnO_3_ is smaller, which is consistent with the experimentally observed reductions in the electrical resistivity and absolute Seebeck coefficient.

**Figure 8:**
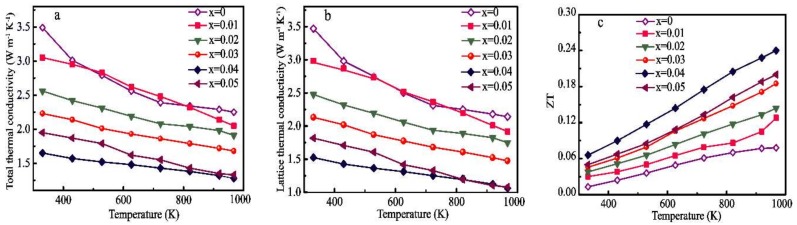
Temperature dependence of (**a**) total thermal conductivity, *κ*_total_; (**b**) lattice thermal conductivity, *κ*_L_; and (**c**) ZT for all the samples.

Figure 8a presents the temperature dependence of the total thermal conductivity (*κ*_total_) in all the samples, which tends to decrease with increasing Pr and Yb dual-doping concentrations. In addition, the *κ*_total_ values of the samples produced through the coprecipitation process are much lower than those of the single- and dual-doped samples synthesized by the solid-state process. The decrease in thermal conductivity can be ascribed to enhanced grain boundary scattering. The phonon scattering at the interfaces of the fine-grained samples is strong, which can significantly decrease the thermal conductivity relative to that of the CaMnO_3_ synthesized by the solid-state process. In general, *κ*_total_ will be presented as the sum of the electron contribution (*κ*_e_) and the lattice contribution (*κ*_L_) [45]. κe can be estimated from the Wiedemann–Franz law (*κ*_e_ = LT/*ρ*), in which L_0_ refers to the Lorentz constant (L_0_ = 2.45 × 10^−8^ W·Ω·K^−2^). As a result of the decreased electrical resistivity, *κ*_e_ increases with increasing Pr and Yb dual-doping concentrations. According to the Wiedemann–Franz law, the estimated values of *κ*_e_ are quantitatively small throughout the temperature range and are less than 20% of *κ*_total_. Therefore, the contribution of *κ*_e_ to *κ*_total_ is negligible, whereas *κ*_L_ dominates the decrease in the thermal conductivity of the lattice, showing an effective method to improve the TE properties of the perovskite CaMnO_3_. The changes in *κ*_L_ as a function of temperature are presented in Figure 8b. From Figure 8b, it can be observed that the lattice thermal conductivity decreases with increasing dual-doping amounts due to the enormous mass difference between the substituting Pr and Yb elements and the matrix element Ca [46] along with the increase in MnO_6_ distortion. The MnO_6_ distortion consequently induces structural changes in the overlap place between the O 2p and Mn 3d orbitals. The change in the electronic structure shown above modifies the thermal transport properties of the Pr and Yb dual-doped CaMnO_3_ series [47].

Figure 8c shows a plot of the ZT value calculated by using the electrical and thermal transport properties over a temperature range of 300–1000 K. The ZT values of all the samples increase as the temperature increases as a result of the two significant decreases in both *ρ* and *κ*_total_. The Pr and Yb dual-doped samples exhibit significantly better performance than the pristine CaMnO_3_. In addition, a certain dual-doping concentration (x = 0.04) has the largest ZT of 0.24, which is several times larger than that of the undoped compounds. However, the ZT value of the Ca_0.92_Pr_0.08_MnO_3_ and Ca_0.90_Yb_0.10_MnO_3_ prepared by the solid-state method is only 0.16 [13,17].

## 4. Discussion

A series of Ca_1−2x_Pr_x_Yb_x_MnO_3_ (x = 0, 0.01, 0.02, 0.03, 0.04 and 0.05) were synthesized through the coprecipitation process. The microstructure, electron transport properties and thermal properties of all the samples were studied. The refinement results indicate that the lattice parameters and cell volumes of dual-doped samples increase with increasing Pr and Yb dual-doping content, indicating that Mn^3+^ is formed in the Mn^4+^ matrix. Moreover, the fact that the increase of the Mn^3+^/Mn^4+^ ratio lead to an increasing in the carrier concentration. This leads to decreased electrical resistivity and a reduced Seebeck coefficient, leading to a maximum PF of 3.48 × 10^−4^ W· m^−1^· K^−2^ at 773 K for the composition x = 0.04. Our first-principles calculations of the DOS support the experimental findings in terms of the band gap and electrical transport properties. The dual-doping samples prepared by the coprecipitation method possess fine grain size, increasing the scattering of grain boundaries, leading to decreased thermal conductivity for the dual-doped samples. Moreover, structural distortion for MnO_6_ along with the enormous mass difference between the dual-doping ions and the calcium ions is attributed to the reduction in the thermal conductivity. It was calculated that the highest dimensionless ZT is 0.24 under the condition of 973 K in the air for Ca_0.92_Pr_0.04_Yb_0.04_MnO_3_, and it is 3 times higher than that of the undoped CaMnO_3_. As a result, the combination of coprecipitation and doping of Pr and Yb at the Ca site is confirmed to effectively promote the TE properties of the CaMnO_3_.

## Figures and Tables

**Table 1:** Refined structure parameters, bond distances, distortion angles, and tolerance factor (t) of the Ca_1−2x_Pr_x_Yb_x_MnO_3_ series.

Ca_1−2x_Pr_x_Yb_x_MnO_3_	x = 0	x = 0.01	x = 0.02	x = 0.03	x = 0.04	x = 0.05
a (Å)	5.2808(2)	5.2833(2)	5.2866(2)	5.2894(2)	5.2931(4)	5.2981(4)
b (Å)	7.4570(3)	7.4595(4)	7.4619(4)	7.4622(3)	7.4662(6)	7.4698(6)
b/$\sqrt{2}$	5.2737	5.2754	5.2771	5.2774	5.2802	5.2827
c (Å)	5.2682(2)	5.2701(2)	5.2728(3)	5.2754(2)	5.2788(4)	5.2815(4)
V (Å^3^)	207.45	207.70	208.0	208.22	208.62	209.02
Rw	11.1	11.0	10.4	12.7	11.7	13.5
Rp	7.39	7.44	6.87	8.26	7.64	8.89
χ^2^	1.74	1.78	1.68	2.63	2.32	3.22
Mn-O2 × 2 (Å)	1.8708(2)	1.8716(2)	1.8728(2)	1.8737(4)	1.8750(4)	1.8765(4)
Mn-O1 × 2 (Å)	1.9110(2)	1.9117(1)	1.9123(2)	1.9125(2)	1.9135(2)	1.9144(2)
Mn-O1 × 2 (Å)	1.9281(2)	1.9289(2)	1.9300(2)	1.9309(4)	1.9322(4)	1.9335(4)
Mn-O1-Mn (°)	158.077(1)	158.078(1)	158.080(1)	158.084(1)	158.085(1)	158.087(1)
Mn-O2-Mn (°)	154.594(1)	154.593(1)	154.589(1)	154.578(1)	154.575(1)	154.573(1)
T	1.000	0.9982	1.9967	1.9950	0.9933	0.9916

**Table 2:** Chemical composition (nominal and measured), and density (dt and da) of Ca_1−2x_Pr_x_Yb_x_MnO_3_ (x = 0, 0.01, 0.02, 0.03, 0.04, and 0.05).

Nominal Composition (x)	Pr Content by ICP	Yb Content by ICP	dt (g/cm^3^)	da (g/cm^3^)	Relative Density (%)
0	-	-	4.58	3.62	79
0.01	0.0089	0.0078	4.64	4.08	85
0.02	0.0179	0.0187	4.71	4.10	87
0.03	0.0285	0.0282	4.78	4.30	89
0.04	0.0388	0.0386	4.84	4.21	90
0.05	0.0487	0.0483	4.92	4.38	89
